# ParaHaplo 3.0: A program package for imputation and a haplotype-based whole-genome association study using hybrid parallel computing

**DOI:** 10.1186/1751-0473-6-10

**Published:** 2011-05-24

**Authors:** Kazuharu Misawa, Naoyuki Kamatani

**Affiliations:** 1 Research Program for Computational Science, Research and Development Group for Next-Generation Integrated Living Matter Simulation, and Fusion of Data and Analysis Research and Development Team, RIKEN, 4-6-1 Shirokane-dai, Minato-ku, Tokyo 108-8639, Japan; 2 Laboratory for Statistical Analysis, RIKEN Center for Genomic Medicine, Tokyo, Japan

**Keywords:** ParaHaplo, haplotype reconstruction, genotype imputation, parallel computing, HapMap, GWAS

## Abstract

**Background:**

Use of missing genotype imputations and haplotype reconstructions are valuable in genome-wide association studies (GWASs). By modeling the patterns of linkage disequilibrium in a reference panel, genotypes not directly measured in the study samples can be imputed and used for GWASs. Since millions of single nucleotide polymorphisms need to be imputed in a GWAS, faster methods for genotype imputation and haplotype reconstruction are required.

**Results:**

We developed a program package for parallel computation of genotype imputation and haplotype reconstruction. Our program package, ParaHaplo 3.0, is intended for use in workstation clusters using the Intel Message Passing Interface. We compared the performance of ParaHaplo 3.0 on the Japanese in Tokyo, Japan and Han Chinese in Beijing, and Chinese in the HapMap dataset. A parallel version of ParaHaplo 3.0 can conduct genotype imputation 20 times faster than a non-parallel version of ParaHaplo.

**Conclusions:**

ParaHaplo 3.0 is an invaluable tool for conducting haplotype-based GWASs. The need for faster genotype imputation and haplotype reconstruction using parallel computing will become increasingly important as the data sizes of such projects continue to increase. ParaHaplo executable binaries and program sources are available at http://en.sourceforge.jp/projects/parallelgwas/releases/.

## Background

Recent advances in various high-throughput genotyping technologies have allowed us to test allelic frequency differences between case and control populations on a genome-wide scale [1]. Genome-wide association studies (GWASs) are used to compare the frequency of alleles or genotypes of a particular variant between cases and controls for a particular disease across a given genome [2-4]. More than a million single nucleotide polymorphisms (SNPs) are analyzed in SNP-based GWASs and haplotype-based GWASs [5,6].

By modeling the patterns of linkage disequilibrium in a reference panel, genotypes not directly measured in the study samples can be imputed [7]. SNP genotype imputation has been proposed as a powerful means to include genetic markers into large-scale disease association studies without the need to actually genotype them [8,9]

To quickly conduct GWASs, we developed a software package for the parallel computation of genotype imputation and haplotype reconstruction called ParaHaplo 3.0. ParaHaplo 3.0 contains all of the functions of ParaHaplo 1.0 [5] and ParaHaplo 2.0 [6], plus it can conduct genotype imputation and haplotype reconstruction using MACH 1.0 [10]. ParaHaplo 3.0 is based on the principle of data parallelism, a programming technique used to split large datasets into smaller ones that can be run in a parallel concurrent fashion [11]. ParaHaplo 3.0 is intended for use in workstation clusters using the Intel Message Passing Interface (MPI).

Using ParaHaplo 3.0, we estimated haplotypes using the genotype data of the Japanese from Tokyo (JPT) and the Han Chinese from Beijing (CHB) obtained from the HapMap dataset [12,13]. Using ParaHaplo 3.0, we compared the speed of haplotype estimation using parallel computation to the number of processors.

## Methods

### Software overview

ParaHaplo supports the genotype data in the HapMap format [14] and the BioBank Japan format [15]. ParaHaplo 3.0 requires an input file of haplotype block boundaries. ParaHaplo 3.0 can conduct genotype imputation and haplotype reconstruction using MACH 1.0 [10]. ParaHaplo 3.0 can also conduct haplotype estimation using PHASE 2.1 [16] and SNPHAP 1.3.1 [17] algorithms. By using hybrid MPI + OpenMP parallelization [18], ParaHaplo 3.0 can conduct haplotype-based GWAS faster than previous versions.

### Parallel computing using MPI methods

ParaHaplo 3.0 is implemented in an MPI-C multithreaded package. The MPI package allows us to construct parallel computing programs on multiprocessors. The genome-wide polymorphism data is broken down into user-defined haplotype blocks, and the MPI Bcast function is used to distribute a single block of haplotype data into each processor. Each processor executes Mach 1.0 [10] and conducts genotype imputation and haplotype reconstruction of a single linkage disequilibrium (LD) block. Once the haplotypes of each LD block are completely estimated, the results are compiled into a single genome-wide dataset through use of the MPI-Gatherv function. ParaHaplo 3.0 is compatible with OpenMPI 1.2.5 and MPICH 1.2.7p1. Users can compile the source code using a GCC compiler, an Intel C compiler, or a Fujitsu C compiler, so that Haplotype-based GWAS can be run on Linux-based PC clusters as well as on K computer (http://www.fujitsu.com/global/news/pr/archives/month/2009/20090717-01.html).

### Hardware

A PC cluster at RIKEN Integrated Cluster of Clusters (RICC) was used when the computational time was measured. The program was compiled using an Intel C compiler. The numbers of processing units used included 1, 2, 4, 8, 16, 32, and 64.

### Example data

An example GWAS is presented here. We used ParaHaplo 3.0 to compare genome-wide genotype data of JPT and CHB from HapMap 3.0 [13]. Some individuals were excluded because they contain too many untyped SNPs. As the reference panel, we used 20,086 SNPs of 170 people. JPT data set was used to be imputed. JPT data set consisted of 82 people with 2,392 SNPs being untyped. Haplotype blocks were obtained as LD blocks using the method outlined by Gabriel *et al*. [19] and the Haploview program [20]. The entire JPT and CHB genomes were divided into 106,149 haplotype blocks by Haploview [20]. Among them, 1,536 haplotype blocks were on chromosome 22.

## Results and discussion

### Genotype Imputation and Haplotype Reconstruction of JPT and CHB

**Figure 1:**
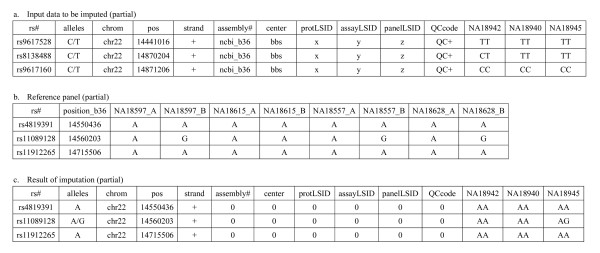
**Results of genotype imputation by ParaHaplo 3.0**. a. Input data to be imputed. From the 1^st ^to 11^th ^columns are the same as HapMap data format. b. Reference panel. Reference panel must be phased. c. Imputed data. From the 1^st ^to 11^th ^columns are the same as HapMap data format.

Figure 1 shows the input and the output data of haplotype phasing. Each line corresponds to a SNP site. From the 1^st ^to 11^th ^columns are the same as HapMap data format. For example, the 1^st ^column shows the rs number of each SNP. From the 12^th ^column to the end of the line, each column corresponds to the genotype at the SNP site of one individual. Figure 1a shows an example of the input data to be imputed. From the 1^st ^to 11^th ^columns are the same as HapMap data format. Figure 1b shows reference panel. Reference panel must be phased by using ParaHaplo 2.0 [6] or by other programs. Figure 1c shows the result of imputation. From the 1^st ^to 11^th ^columns are the same as HapMap data format. Columns without any information are filled by 0.

### Calculation Time

Table 1 shows the elapsed times and the speedups associated with the use of ParaHaplo 3.0 using the genotype data of chromosome 22 for haplotype estimation. The speedup ratio is the ratio of the computation time of a single processor to that of multiple processors. As shown in Table 1 the calculation time decreased as the number of processors increased. When 64 processors were used, ParaHaplo functioned 20 times faster than the non-parallel program.

**Table 1:** Elapsed times and speedups obtained using ParaHaplo 3.0 in the imputation process

Number of Processing Units	Elapsed Time			**Speed Ratio**^a^
1	4 h	21 m	59 s	1
2	2 h	12 m	2 s	1.98
4	1 h	10 m	3 s	3.74
8		38 m	46 s	6.76
16		24 m	30 s	10.69
32		13 m	25 s	19.51
64		11 m	25 s	22.94

^a^Ratio of computation time of a single processor to computation time of multiple processors

### Parallel Computation of Haplotype-Based GWAS

The results show that the parallel computing ability of ParaHaplo 3.0 for haplotype estimation was 20 times faster than that of the non-parallel version of ParaHaplo 3.0. In this study, we used a total of 89 JPT and CHB individuals whose genotypes had been determined during the HapMap project [12]. When a single processor was used, haplotype estimation for chromosome 22 took more than 4 h; if 9,000 individuals were to be analyzed under the same conditions, the analysis would take more than 2 weeks. However, if ParaHaplo 3.0 was used on a workstation with 64 processors, the same analysis would take less than 1 day. However, the relatively lower speed ratio could be affected by the inflation of sample size [11]. Further study is required.

Even when 64 processors were used, the speedup ratio was only 22 because of the variations in the LD block size. ParaHaplo is based on data parallelism, and our result showed that the computation time of each genotype imputation was approximately proportional to the number of SNPs within the LD block (data not shown); therefore, we believe that a large LD block may create a computational bottleneck as does in haplotype estimation [6].

## Conclusions

We developed ParaHaplo 3.0, a set of computer programs, for the parallel computation of haplotype estimation and accurate P values in haplotype-based GWASs. ParaHaplo is intended for use in workstation clusters using the Intel MPI. Using ParaHaplo, we conducted haplotype estimation of JPT and CHB genotype data taken from the HapMap 3.0 dataset [12].

These results indicate that when the number of processors is sufficient, the parallel computing abilities of ParaHaplo are 20 times faster than those of non-parallel programs. Accurate and complete genotypes have been obtained for more than a million SNPs [15], and >10,000 individuals are now being genotyped [21]. The need for fast haplotype estimation using parallel computing will become increasingly important as project data sizes continue to increase.

## Availability and Requirements

•**Project name: **ParaHaplo 3.0

•**Project home page: **http://sourceforge.jp/projects/parallelgwas/releases/46982

•**Operating systems: **Platform independent

•**Programming language: **Java and C

•**Other requirements: **OpenMPI version 1.2.5, or MPICH version 1.2.7p1

•**License: **MIT license

•**Any restrictions for use by non-academics: **License required

## List of abbreviations

GWAS, Genome-Wide Association Study; SNP, Single Nucleotide Polymorphism; LD, Linkage Disequilibrium; RAT, Rapid Association Test; SPT, Standard Permutation Test; MCMC, Markov-chain Monte Carlo; JPT, Japanese Tokyo; CHB, Han Chinese Beijing

## Competing interests

The authors declare that they have no competing interests.

## Authors' contributions

KM designed the software and wrote the manuscript, and NK supervised the project. Both authors have read and approved the final manuscript.
